# P08-08 Exploring paths of physical activity for city residents using mobile applications and geographic information system

**DOI:** 10.1093/eurpub/ckac095.121

**Published:** 2022-08-29

**Authors:** Amir Duhair, Yaqouta Ghebghoub

**Affiliations:** 1 Faculty of sciences and technology, university of jijel, jijel, Algeria; 2 Faculty of Economics, Commerce and Management Sciences, university of jijel, jijel, Algeria

**Keywords:** GIS, city residents, smart surroundings, mobile applications

## Abstract

**Background:**

The built environment is one of the main places where residents engage in their physical activity. Therefore, it is important to identify the track most frequently used by the population. This was done by designing a mobile application that charts the track that residents take when practicing their sports and sends them to a specially designed data hall to arrange and classify information. Since there are no tracks dedicated to exercising in the Jijel city, Algeria; the study aimed to identify the most frequently used tracks, as well as identify the points that constitute an obstacle in those tracks.

**Methods:**

During April-May 2021, 107 people (76 men, 31 women) from city of Jijel (Algeria) participated in the study (Purposive Sample). Participants were provided with a mobile application (APK file) linked to a database that collects and arranges data and mapped with ArcGIS software. The experiment lasted for 30 consecutive days. Where the application was programmed to start automatically at 06:00 am to 09.00 am. The application maps participants' track, and identifying places where deceleration occurs.

**Results:**

Around eighty-two percent (82.2%) chose three main tracks, two coastal tracks (A,B), and a mountain track (C), These are the paths that have been analysed and studied. A total of 2552 hours of data were collected, with an average of 29 hours per person. In the three tracks, there were 18 sub-tracks (less than 10 meters), These sub-paths are distributed as (A:06, B:09, C:03). The coastal tracks (A,B) witnessed a noticeable slowdown in movement speed. This slowdown is related to the presence of obstacles such as unpaved roads, congested traffic points and narrow pedestrian paths, which witness congestion in the morning. The mountain track (C) considered the least slowed path due to the absence of traffic congestion.

**Conclusions:**

The tracks and the three lanes must be rehabilitated in a way that allows residents to engage in physical activity in a smooth and safe manner. Some changes have been suggested in the lanes and marked with signs dedicated, with a recommendation to intervene at 43 obstacles.

